# Sequence of Antibiotic Administration for the Treatment of *Mycobacterium avium* subsp. *hominissuis* Infection Might Influence the Response Outcome

**DOI:** 10.3390/antibiotics15060593

**Published:** 2026-06-10

**Authors:** Amy Leestemaker-Palmer, Stephanie Nuss, Luiz E. Bermudez

**Affiliations:** 1 Department of Biomedical Sciences, Carlson College of Veterinary Medicine, Oregon State University, Corvallis, OR 97331, USA; amy.palmer@oregonstate.edu (A.L.-P.); stephanie.nuss@oregonstate.edu (S.N.); 2 Department of Microbiology, College of Sciences, Oregon State University, Corvallis, OR 97331, USA

**Keywords:** ethambutol, clarithromycin, rifabutin, *Mycobacterium avium* subsp. *hominissuis*, biofilm, macrophages, inhibition, killing

## Abstract

Background/Objectives: *Mycobacterium avium* subsp. *hominissuis* is a cause of disseminated infections in immunosuppressed patients and pulmonary infections in individuals with chronic lung conditions. The treatment of this infection is prolonged, and ultimately associated with failure in a large percentage of patients. Previous studies have determined that the bacteria respond to antibiotic contact, altering pathways affecting metabolic and other functions, as well as structural aspects. Methods: Based on this assumption and the fact that the medication used to treat *M. avium* infection may encounter the target bacterium at different sequential times, we evaluated the effect of three antibiotics, clarithromycin, ethambutol and rifabutin, delivered together or separately in different orders. Results: The results showed no difference regarding the response of the bacterium to treatment with the three antibiotics when in planktonic state. However, in either the biofilm phenotype or inside macrophages, different orders of exposure to antibiotics led to different responses. In macrophages, the sequence of exposure when ethambutol was given first, followed by clarithromycin and rifabutin, was associated with bacterial count decrease, similar to the simultaneous exposure to the three antibiotics, whereas the other combinations showed a decreased effect. Conclusions: In summary, the study highlights an important aspect of bacterial response to combination therapies, and more specifically, the response of *M. avium* to therapy. The use of a cell wall-active antibiotic first appears to be linked to an improvement in therapy efficacy.

## 1. Introduction

Infections caused by *Mycobacterium avium* subsp. *hominissuis* (hereafter referred to as *M. avium*) are currently a common cause of respiratory infection in individuals with chronic pulmonary conditions such as cystic fibrosis, non-cystic fibrosis bronchiectasis, and emphysema [1,2]. The bacterium is encountered in the environment, mainly in soil and water [3,4], which are assumed to be the source of the majority of infections. Recently, however, *M. avium* and *Mycobacterium abscessus* have also been shown to be transmitted from patient to patient [5]. This observation has been reinforced by experiments showing that bacteria can move between hosts without environmental contact [6,7].

*M. avium* are a heterogeneous group of bacteria that express resistance to most available antibiotics [8,9]. Their natural resistance is made more significant by the prolonged period of treatment, which commonly leads to the appearance of resistant or tolerant phenotypes, resulting in the failure of therapy.

The treatment of infections caused by other pathogens in patients with predisposing conditions also facilitates the development of phenotypic tolerance [10,11], due to the long period of time associated with therapy. The treatment of *M. avium* infection involves the use of at least three antibiotics, in which clarithromycin or azithromycin are used in combination with ethambutol for recommended initial therapy [12]. Rifampin or rifabutin complement the employed therapeutic regimen. Because of patient tolerance to antibiotics, the therapeutic regimen initially provides an interval of approximately a week between the antibiotics in the regimen.

Recent studies with *M. avium*, *M. abscessus*, and *Pseudomonas aeruginosa* have demonstrated that bacteria exposed to antibiotics can react by altering the metabolic pathways, cell wall or cell membrane, in an attempt to adapt to the presence of the drugs been used [13,14]. The exposure to a bacteriostatic or bactericidal concentration of an antibiotic triggers a response from the bacteria, often involving changes in membrane permeability or a “metabolic escape” [15]. For example, when exposed to antibiotics under environmental conditions mimicking the lung, *M. avium* synthesize large amounts of LprB lipoprotein, which in *Mycobacterium tuberculosis* has been associated with virulence and in *M. avium* has been linked to drug resistance [16,17]. In *M. abscessus*, the exposure to amikacin induces the synthesis of peptidoglycan, as well as fatty acid biosynthetic pathways, an indication of the attempt by the bacterium to modify the cell wall, consequently influencing the susceptibility to antibiotics. This response occurs unrelated to the duplication time of the bacterium.

Therefore, the exposure to an antimicrobial has the potential to modify the pathogen prior to being reached by the next antibiotic. These above-cited examples created the hypothesis that combination therapies used to treat mycobacterial infections in patients do not reach the bacterial target within the same timeframe. This allows for the alteration of the target bacterium, making the order in which the antibiotics are administered influential to the overall response to the therapy. In fact, different antibiotics have diverse PK/PD and tissue distribution. While rifampin has a volume distribution of 0.65 L/kg, t_1/2_ = 1.5 to 5 h and AUC of 40–60 μg/mL, clarithromycin has a vol distribution of 4 L/kg, t_1/2_ = 4–5 h and AUC of 20 μg/mL, and ethambutol has a volume of distribution of 6 L/kg, t_1/2_ = 4 h and AUC of 29.6 μg/mL. Therefore, the antibiotic molecule will likely encounter the bacterium at diverse timepoints, which is more realistic from a clinical perspective.

In this study, we question whether the order of administration of antibiotics could result in a decrease in the anti-bacterial effect of the combination. Our results show that exposure to the combination of antibiotics administered either together or separated by a time interval did not affect the bacterial response of planktonic bacteria; the treatments were equally effective. Although an anti-bacterial response was observed in both the macrophage environment and in biofilm; the effect varied dependent of the order of antibiotics given as the therapy. Because biofilm formation and infection of macrophages are part of the strategy used by *M. avium* in the host lung, the findings suggest a plausible clinical implication of the sequencing of antibiotic administration.

## 2. Materials and Methods

### 2.1. Bacteria Maintenance and Tissue Culture

*Mycobacterium avium* subsp. *hominissuis* strain 104 (*M. avium* 104) was obtained from the American Type Culture Collection (ATCC). *Mycobacterium avium* strain 100 was obtained from the Dr. Inderlied collection at Los Angeles Children’s Hospital and has been previously described [18]. *M. avium* 104 and 100 were grown on Middlebrook 7H10 agar supplemented with 10% *w*/*v* oleic acid, albumin, dextrose, and catalase (OADC, Hardy Diagnostics, Santa Maria, CA, USA) for 7–10 days at 37 °C. For the assays, bacteria were suspended in Middlebrook 7H9 broth to the desired concentrations, spun down, and washed, and the final inoculum was established in Hanks Balanced Salt Solution (HBSS). *M. avium* 104 and 100 biofilms were established by seeding 1 × 10^7^ bacteria per well in a 96-well tissue culture plate diluted from an inoculum of 3 × 10^8^ CFU/mL (1 McFarland standard). Biofilms matured for 7 days at 37 °C before antimicrobial treatment.

THP-1 (TIB-202) human monocytes were obtained from the ATCC and maintained in RPMI 1640 supplemented with 10% heat-inactivated fetal bovine serum (FBS; Gemini Bio-Products, West Sacramento, CA, USA) at 37 °C with 5% CO_2_. THP-1 cells were differentiated with 50 ng/mL of Phorbol 12-myristate 13-acetate (PMA; Sigma Aldrich, St. Louis, MO, USA) for 24 h, followed by 24 h in media without PMA, prior to use in experiments.

### 2.2. Minimum Inhibitory Concentration and Minimum Bactericidal Concentration Determination

*M. avium* 104 was used to determine MICs and MBCs of rifabutin, clarithromycin, and ethambutol. A range from a high dose to a low dose of antimicrobials was used to determine bacteria susceptibility. *M. avium* 104 was grown to log phase (7 days) on 7H10 Middlebrook media plates supplemented with 10% OADC. For each antimicrobial, 128 µg/mL was added to 2 mL of 7H9 broth supplemented with OADC then diluted 1:1 until the concentrations reached 0.125 µg/mL. *M. avium* 100, as previously shown, has a MIC smaller than that of *M. avium* 104. Control tubes containing no antimicrobials were also included for each antimicrobial tested. Inoculums of 10^9^ bacteria were measured with an optical density (O.D.) of 595 nm. Bacteria was added to each antimicrobial tube at 3 × 10^5^ CFU/mL and incubated in a shaking incubator at 37 °C and 200 rpm. After 10 days of incubation, 100 µL from each tube was placed in a 96-well plate for O.D. reading. Turbidity determined sensitive, intermediate, or resistant bacteria. Minimum bactericidal concentrations were determined by plating broth with antimicrobials from the MIC experiment to determine whether growth occurred with the removal of the antimicrobial and at what concentration bacteria was killed [19]. The results are summarized below in Table 1.

### 2.3. Planktonic Bacteria Sequential Treatment with Antimicrobials

*M. avium* 104 and 100 were grown to log phase (7 days) on 7H10 Middlebrook media plates supplemented with 10% OADC as previously described [20]. *M. avium* has a division time in vitro of approximately every 12–16 h. Although a much shorter interval of time was chosen for treatment, it was based on past results, which have shown that the moment an antibiotic comes into contact with bacteria (mycobacteria included), the bacteria begin to respond [13,14,15]. A bacteria inoculum of 3 × 10^8^ CFU/mL was made in 7H9 broth; then, 3 × 10^5^ CFU/mL was added to 5 mL culture tubes containing each of the antimicrobial treatments. Each antimicrobial tested was solubilized and diluted to working stocks in dH_2_O (rifabutin: 100 µg/mL, clarithromycin: 250 µg/mL, and ethambutol: 500 µg/mL). Testing of antimicrobial combinations with a time delay were as follows: clarithromycin + rifabutin + ethambutol (CRE); clarithromycin + ethambutol + rifabutin (CER); rifabutin + clarithromycin + ethambutol (RCE); rifabutin + ethambutol + clarithromycin (REC); ethambutol + rifabutin + clarithromycin (ERC); ethambutol + clarithromycin + rifabutin (ECR). Antimicrobials were added to culture tubes at concentrations determined by MICs summarized in Table 1. Each sequential treatment had a 15 min or 30 min time delay before the addition of the next antimicrobial in the sequence. A positive control of clarithromycin + rifabutin + ethambutol was added to bacterial suspension without time delays (same). A negative (−) control of no antimicrobial treatment was used to determine bacterial growth for comparisons. After completion of the antimicrobial sequence, bacteria was incubated at 37 °C on a shaking incubator at 200 rpm. Bacteria growth was determined on day 1, day 5, and day 7 by serially diluting the treatment bacterial suspension to quantify colony forming units (CFUs).

### 2.4. Biofilm Sequential Treatment with Antimicrobials

*M. avium* 104 and 100 biofilms were seeded 1 × 10^5^ bacteria per well in a 96-well tissue culture plate then matured for 7 days at 37 °C before antimicrobial treatment, as previously described [21]. Each antimicrobial tested was solubilized and diluted to working stocks in HBSS (rifabutin: 100 µg/mL, clarithromycin: 250 µg/mL, and ethambutol: 500 µg/mL). Testing of antimicrobial combinations with a time delay was the same as described above. Antimicrobials were added to biofilms at concentrations determined by the MICs summarized in Table 1. Each sequential treatment had a 5 min or 15 min time delay before the addition of the next antimicrobial in the sequence. A positive control of the three antimicrobials was added to biofilms without time delays (same). A negative (−) control of no antimicrobial treatment was used to determine biofilm robustness. Briefly, the biofilm supernatant was removed and HBSS containing each antimicrobial was added at their respective times. After completion of the antimicrobial sequence, biofilms were incubated at 37 °C. Bacteria was quantified after 10 days by serially diluting the treatment bacterial suspension to quantify colony forming units (CFUs).

### 2.5. Electron Microscopy

Biofilm samples were fixed in 2.5% glutaraldehyde and 1% paraformaldehyde in 0.1 M sodium cacodylate buffer and dehydrated with increasing concentrations of ethanol. Once in 100% ethanol, samples were dropped into liquid nitrogen and cracked by placing them on a piece of metal in the liquid nitrogen, laying a razor blade over each piece, and gently tapping the blade with a hammer. The cracked pieces were placed in 100% ethanol to come to room temperature, and then critical point dried. Samples were mounted on stubs with the newly exposed cracked surface visible to the scanning electron microscope (SEM) beam, and were imaged on a Quanta 600F SEM (Thermo Fisher, Hillsboro, OR, USA).

### 2.6. Macrophage Sequential Treatment with Antimicrobials

THP-1 cells were differentiated with 50 ng/mL of PMA for 24 h, followed by 24 h in whole RPMI 1640 with 10% FBS media without PMA, prior to use in experiments [19]. Differentiated THP-1 macrophages were infected with *M. avium* 104 or *M. avium* 100 for 1 h with a MOI of 5. Infected cells without antimicrobial treatment were used as a negative control. Cells were washed three times to remove extracellular bacteria, and non-internalized bacteria were killed by incubating the monolayers with 100 mg/mL of amikacin for 2 h. Then, monolayers had the supernatant removed, were washed with HBSS once, and RPMI-1640 supplemented with 10% FBS was added. Each antimicrobial tested was solubilized and diluted to working stocks in RPMI 1640 (rifabutin: 100 µg/mL, clarithromycin: 250 µg/mL, and ethambutol: 500 µg/mL). Testing of antimicrobial combinations with a time delay was performed in the same way as described above. Antimicrobials were added to biofilms at concentrations determined by MICs summarized in Table 1. Each sequential treatment had a 5 min, 15 min and 30 min time delay before the addition of the next antimicrobial in the sequence. A positive control of the three antimicrobials was added to biofilms without time delays (same). Survival of *M. avium* 104 and 100 in THP-1s were determined 5 days post infection. At the indicated timepoints, cell media was removed and replaced with 400 μL of H_2_O for lysis. Wells were pipetted 25 times to disrupt cells then diluted and plated for CFU enumeration.

### 2.7. Statistical Analysis

To evaluate the significance of the observation, statistical analysis was performed by using GraphPad Prism version 8 software (Boston, MA, USA). ANOVA was used to evaluate the significance for multiple comparisons. Differences were considered significant at *p* < 0.05.

## 3. Results

### 3.1. Efficacy of Antimicrobials in a Time-Dependent Manner Against M. avium in 7H9 Culture Broth

Antimicrobials act on microorganisms through various mechanisms, and utilizing these can lead to treatment breakthroughs. Combinations of antimicrobials can behave synergistically, antagonistically, or neutrally and are a concern for individuals who need a multidrug strategy for infection treatments. First, we investigated whether treating *M. avium* with three antimicrobials in a time-dependent manner would improve bacteria-killing properties compared to administering the three antimicrobials at the same time. Minimal inhibitions concentrations were utilized for downstream assays (Table 1).

The intervals between exposure to antibiotics are exploratory settings and do not precisely represent clinical settings, where many influential factors may be present.

**Figure 1:**
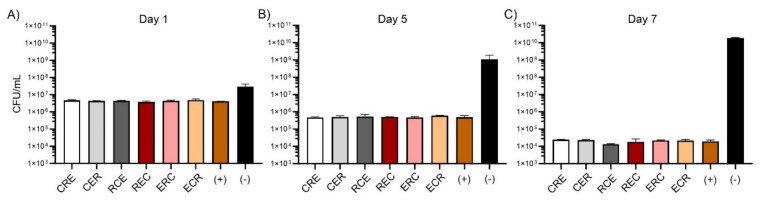
Bactericidal effects of sequential antimicrobial treatment. *M. avium* 104 culture was treated with a sequential dose of three antimicrobials, clarithromycin (C), rifabutin (R), and ethambutol (E). The dosage was determined by MIC, 2 µg/mL (C), 0.25 µg/mL (R), and 8 µg/mL (E). Antimicrobials were given one at a time with a 15 min delay between each addition. The positive control (+) received all three antimicrobials at the same time, while the negative control (−) had no antimicrobial treatment. CFU recovery was determined at day 1 (**A**), day 5 (**B**), and day 7 (**C**) post completion of sequential antimicrobial addition. Graphic representation of three biological replicates.

**Figure 2:**
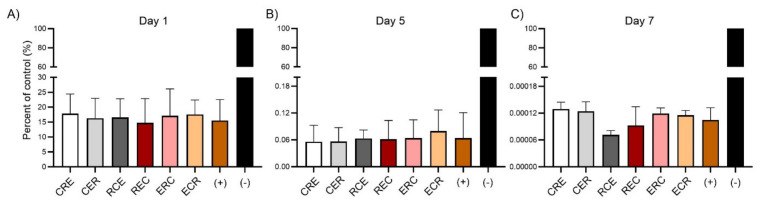
Percentage of viable bacteria after antimicrobial treatment. *M. avium* 104 culture was treated with a sequential dose of three antimicrobials, clarithromycin (C), rifabutin (R), and ethambutol (E). The dosage was determined by MIC, 2 µg/mL (C), 0.25 µg/mL (R), and 8 µg/mL (E). Antimicrobials were given one at a time with a 15 min delay between each addition. The positive control (+) received all three antimicrobials at the same time, while the negative control (−) had no antimicrobial treatment. CFU counts were normalized to the (−) control to determine the percentage of viable bacteria after antimicrobial treatment at day 1 (**A**), day 5 (**B**), and day 7 (**C**). Graphic representation of three biological replicates.

**Figure 3:**
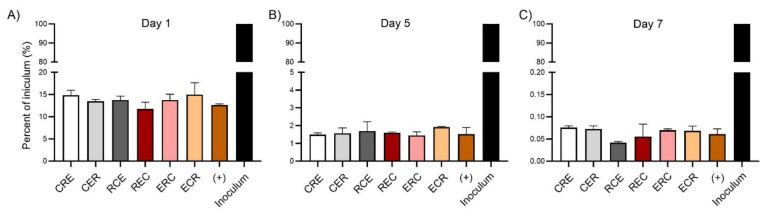
Percentage of viable bacteria after antimicrobial treatment compared to inoculum. *M. avium* 104 culture was treated with a sequential dose of three antimicrobials, clarithromycin (C), rifabutin (R), and ethambutol (E). The dosage was determined by MIC, 2 µg/mL (C), 0.25 µg/mL (R), and 8 µg/mL (E). Antimicrobials were given one at a time with a 15 min delay between each addition. The positive control (+) received all three antimicrobials at the same time. CFU recovery was determined at day 1, day 5, and day 7 post completion of sequential antimicrobial addition. CFU counts were normalized to the inoculum to determine the percentage of viable bacteria after antimicrobial treatment at day 1 (**A**), day 5 (**B**), and day 7 (**C**). Graphic representation of three biological replicates.

Antimicrobial combinations of clarithromycin, rifabutin, and ethambutol were added to 7H9 growth media containing *M. avium* with 15 min intervals between each addition of antimicrobials. CFUs were determined 1, 5, and 7 days post treatment completion with serial dilutions. All combinations of antimicrobials demonstrated similar killing of viable bacteria as the (+) control—all antimicrobials administered at the same time (Figure 1). The (−) control, untreated bacteria, showed growth over 7 days. To determine efficacy between treatment combinations, CFUs were normalized to the untreated control on each day by dividing the CFUs of treatment groups by the CFUs of the untreated control, then multiplying by 100 for the percentage of the control (Figure 2). After 24 h of antimicrobial treatment, all combinations reduced bacteria in broth culture to approx. 15% compared to the untreated control (Figure 2A). All antimicrobial combinations killed bacteria by day 5, leaving approx. 0.06% viable organisms in the culture media compared to the untreated control (Figure 2B). At day 7, viable bacteria in the culture media was approx. 0.00012% that of the untreated control (Figure 2C). There were no significant differences in bacteria killing among any of the antimicrobial combinations over time, unlike the significant reduction in all groups compared to no treatment. Also, we compared all treatment combinations to the initial inoculum to determine efficacy, excluding the untreated growth, which may skew interpretations. CFUs were normalized to the inoculum on each day by dividing the CFUs of treatment groups by the CFUs of inoculum then multiplying by 100 for the percentage of the control (Figure 3). After 24 h of antimicrobial treatment, all combinations reduced bacteria in the broth culture to approx. 15% compared to the inoculum (Figure 3A). All antimicrobial combinations killed bacteria by day 5, leaving approx. 2% viable organisms in culture media compared to the inoculum (Figure 3B). At day 7, viable bacteria in culture media were approx. 0.12% compared to the inoculum (Figure 3C). There were no significant differences in bacteria killing among any of the antimicrobial combinations over time, unlike the significant reduction in all groups compared to the initial inoculum.

**Figure 4:**
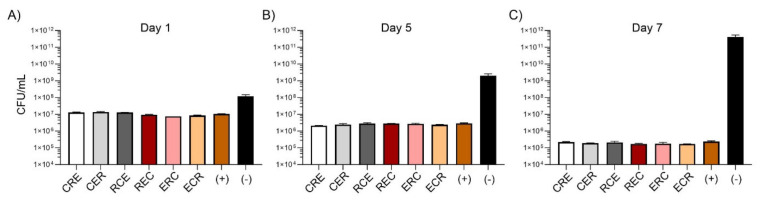
Bactericidal effects of sequential antimicrobial treatment. *M. avium* 104 culture was treated with a sequential dose of three antimicrobials, clarithromycin (C), rifabutin (R), and ethambutol (E). The dosage was determined by MIC, 2 µg/mL (C), 0.25 µg/mL (R), and 8 µg/mL (E). Antimicrobials were given one at a time with a 30 min delay between each addition. The positive control (+) received all three antimicrobials at the same time, while the negative control (−) had no antimicrobial treatment. CFU recovery was determined at day 1 (**A**), day 5 (**B**), and day 7 (**C**) post completion of sequential antimicrobial addition. Graphic representation of three biological replicates.

**Figure 5:**
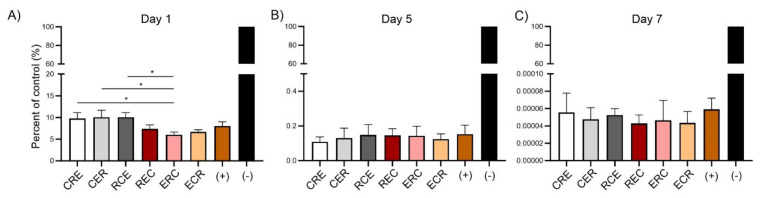
Percentage of viable bacteria after antimicrobial treatment. *M. avium* 104 culture was treated with a sequential dose of three antimicrobials, clarithromycin (C), rifabutin (R), and ethambutol (E). The dosage was determined by MIC, 2 µg/mL (C), 0.25 µg/mL (R), and 8 µg/mL (E). Antimicrobials were given one at a time with a 30 min delay between each addition. The positive control (+) received all three antimicrobials at the same time, while the negative control (−) had no antimicrobial treatment. CFU counts were normalized to (−) control to determine the percentage of viable bacteria after antimicrobial treatment at day 1 (**A**), day 5 (**B**), and day 7 (**C**). Graphic representation of three biological replicates. *p*-value < 0.05 = *.

**Figure 6:**
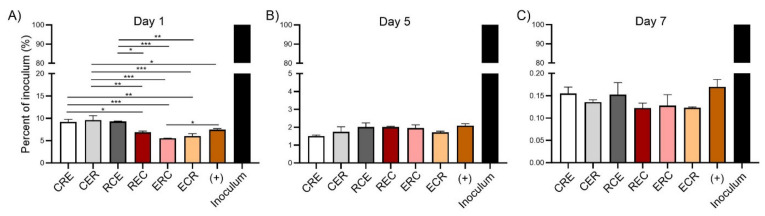
Percentage of viable bacteria after antimicrobial treatment compared to inoculum. *M. avium* 104 culture was treated with a sequential dose of three antimicrobials, clarithromycin (C), rifabutin (R), and ethambutol (E). The dosage was determined by MIC, 2 µg/mL (C), 0.25 µg/mL (R), and 8 µg/mL (E). Antimicrobials were given one at a time with a 30 min delay between each addition. The positive control (+) received all three antimicrobials at the same time. CFU recovery was determined at day 1, day 5, and day 7 post completion of sequential antimicrobial addition. CFU counts were normalized to inoculum to determine the percentage of viable bacteria after antimicrobial treatment at day 1 (**A**), day 5 (**B**), and day 7 (**C**). Graphic representation of three biological replicates. *p*-value < 0.05 = *, *p*-value < 0.1 = **, *p*-value 0.01 = ***.

These experiments were repeated with 30 min of incubation between each antimicrobial addition except the (+) control—all compounds added at the same time—and the (−) control—no treatment. Similarly to the 15 min interval study, CFUs were quantified at day 1, 5, and 7 post antimicrobial treatment (Figure 4). The CFUs were normalized to untreated (−) control bacteria in culture expressed as a percentage of control (Figure 5). All combinations of antimicrobial treatment significantly reduced bacteria in the growth culture to approx. 5–10% of that of the untreated control after 24 h exposure. There was a significant decrease in bacteria treated with ethambutol, rifabutin, then clarithromycin (Figure 5A) compared to CRE, CER, and RCE but not REC, ECR, and (+) control. The ERC treatment had the greatest reduction in bacteria by day 1. All combinations of antimicrobials significantly reduced bacterial load to approx. 0.1% of that of the untreated control, while there was no significant difference observed between antimicrobial treatment groups by day 5 (Figure 5B). Seven days post antimicrobial treatment saw a significant reduction in bacteria to approx. 0.0004% compared to the untreated control, without differences between all antimicrobial treatments (Figure 5C). Again, we normalized efficacy to the initial inoculum to observe bacteria killing. All combinations of antimicrobial treatment significantly reduced bacteria in the growth culture to approx. 5–10% of the inoculum after 24 h exposure. There was a significant decrease in bacteria treated with ethambutol, rifabutin, then clarithromycin (Figure 6A) compared to CRE, CER, RCE, and (+) control but not REC, ECR. There was significant killing of bacteria with ethambutol, clarithromycin, and rifabutin compared to CRE, CER, and RCE, while treatment with all antimicrobials at the same time reduced bacteria significantly compared to CER-only. The ERC treatment had the greatest reduction in bacteria by day 1. All combinations of antimicrobials significantly reduced bacterial load to approx. 2% of the inoculum, while there was no significant difference observed between antimicrobial treatment groups by day 5 (Figure 6B). Seven days post antimicrobial treatment saw a significant reduction in bacteria to approx. 0.2% of the inoculum, without differences between all antimicrobial treatments (Figure 6C).

### 3.2. Efficacy of Antimicrobials in a Time-Dependent Manner Against M. avium Biofilms

**Figure 7:**
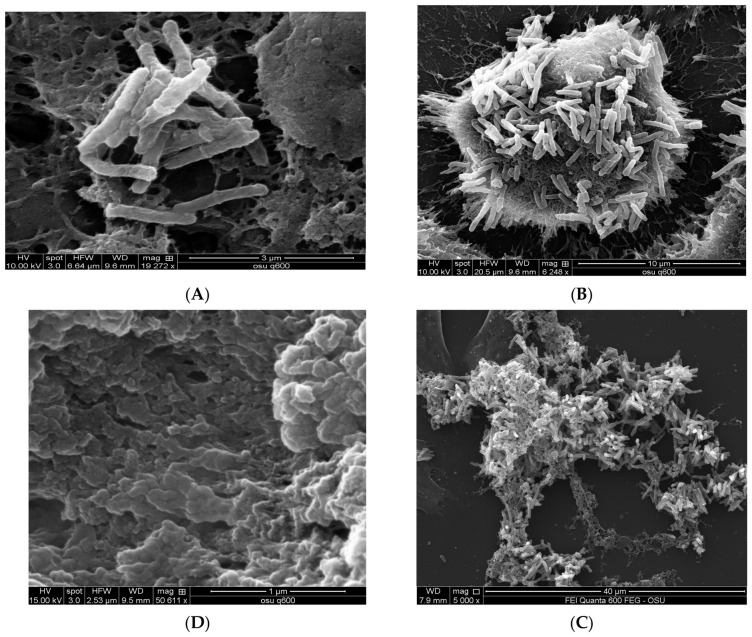
Electronic micrograph of *M. avium* biofilm in different phases of maturation. (**A**) 4 h, (**B**) 24 h, (**C**) 4 days, (**D**) 7 days.

Since *M. avium* can form aggregates that mature into biofilms on the lung surface (Figure 7A–D), we determined whether administering clarithromycin, rifabutin, and ethambutol with intervals between each antimicrobial would improve biofilm clearance. We utilized the same MICs determined previously on planktonic bacteria on established biofilms. These biofilms were exposed to antimicrobial combinations with either 5 or 15 min intervals between each compound addition; then, after 10 days, CFUs were determined by serial dilution, summarized for MAH 104 in Table 2. Only the treatments with ethambutol, clarithromycin, and rifabutin (5 and 15 min) or all antimicrobials given at the same time significantly reduced bacteria recovered from biofilms compared to wildtype. With a 15 min interval, only treatment with ethambutol given first and with all the antimicrobials given at the same time reduced bacteria recovered from biofilms.

### 3.3. Efficacy of Antimicrobials in a Time-Dependent Manner Against M. avium-Infected Macrophages

*M. avium* survival in macrophages is well-known, but antimicrobial compounds interact with eukaryotic cells in different ways, affecting their kinetics. We tested our three compounds against established *M. avium* infections of THP-1 macrophages using the strains MAH 104 and MAH 100, 5 days post infection, with either a 5, 15, or 30 min interval between each compound; the results are summarized in Table 3 and Table 4. At 5 min intervals, no combination was more effective than another and not significantly different than both strains of wildtype *M. avium*. With a 15 min interval, we begin to see a decline in *M. avium* CFUs recovered from macrophages, with only the treatment of compounds given at the same time reducing viable bacteria by 1 log compared to wildtype *M. avium* 104 and 100 strains. Overall, these data indicate that bacteria react to ethambutol, clarithromycin, and rifabutin in different combinations when either planktonic or in biofilms, but once internalized by macrophages, any advantage seen previously diminishes.

## 4. Discussion

Antibiotics are administered to patients to treat infections which occur in different organs. The antibiotic molecules have diverse pharmacokinetics, and therefore reach their targets at different times. Infections for which treatment includes more than one antibiotic, either to achieve an additive or synergistic effect, or to prevent the emergence of resistance of the pathogen, may be associated with a problem that is not very often considered. Antibiotics, once they reach the pathogen, trigger a biological response from the bacterium that may happen quickly [10,15]. Studies have investigated the response of bacteria to antibiotics; however, not in multi-antibiotic treatment conditions. When coming into contact with the bacterium, an antibiotic molecule initiates a rapid response in the pathogen [13,14,15].

Mycobacteria infections are usually treated by using two, three or more drugs [9,12]. These compounds are not administered together, and when given as pills or IM/IV, they have diverse pharmacologic properties, which implies that the antibiotics will reach the bacterial target at different timepoints after ingestion or injection, for example. What was shown in the current study is that coming into contact with the bacteria even a few minutes apart might influence the antibiotics’ ability to affect the course of infection. Previous work of different groups has demonstrated that once the antibiotic touches the bacterial cell, it triggers a metabolic response that in many cases leads to tolerance to the antibiotic [9]. Using *Mycobacterium tuberculosis* as a model system, our group was able to dissect the stages of bacterial response to currently used therapy, which demonstrated the shifts through different pathways in a continuous attempt to survive. Those metabolic shifts had no relationship with the duplication time of the pathogen. The time needed for the killing of the bacteria was approximately 6 days, which created opportunities to establish tolerance to the used therapeutic agent [15].

A similar phenomenon was observed when *M. avium* was exposed to antibiotics at specific environmental conditions, such as aerobic, anaerobic or biofilm. Overall, 4000 proteins were evaluated, with numerous proteins being synthesized “de novo” only on anaerobic and biofilm conditions. Among the enriched proteins, many belonged to pathways of pantothenate and CoA biosynthesis, nitrogen metabolism, glycerolipid metabolism, and chloroalkene degradation, known to be linked to antibiotic tolerance in *M. tuberculosis* when upregulated. *M. avium* over-synthesized LprB lipoprotein (also known as LprG) when under the biofilm phenotype upon antibiotic treatment, a protein that has been associated with virulence in *M. tuberculosis*. When over-expressed in *M. avium*, LprB was shown to induce more tolerance to antibiotics than the wildtype bacteria [16]. The activation of peptidoglycan and fatty acid biosynthesis pathways in both *M. avium* and *Mycobacterium abscessus* when exposed to antibiotics indicates an attempt of these pathogens to modify their cell wall, impacting their susceptibility to antibiotics [13,14].

Our follow-up work suggests that bacterial response to antibiotic treatment is quick, and may often be independent of protein expression. It was interesting to observe that a sequence of administration beginning with ethambutol led to approximately similar efficacy achieved by the treatment with all three antibiotics, clarithromycin, ethambutol and rifabutin, within macrophages and biofilms. In contrast, the results obtained when clarithromycin or rifampin were given as the initial antibiotic were not very remarkable. Although exact explanation is still unknown, it is possible that ethambutol being active against the cell wall was important to allow for the other compounds to be effective. This conclusion, however, is preliminary and would require confirmation in future experiments. Another important result was that the different sequences of exposure did not differ in anti-*M. avium* activity when applied to planktonic bacteria, but only when the pathogen was under a situation of stress, either intracellularly in a phagocyte or in a biofilm phenotype.

The establishment of biofilm is a significant strategy employed by *M. avium* and many other NTMs to create a niche on the mucosal surface. Biofilms are notorious for their the increased resistance to antibiotic treatment, either by preventing antibiotic penetration through the extracellular matrix, or by maintaining a percentage of the bacterial population in a non-replicative state [21]. The results obtained when exposing biofilms to a sequence of drugs suggest that the exposure to ethambutol followed by clarithromycin and rifabutin was more effective than the response to the ethambutol, rifabutin and clarithromycin sequence. The explanation of this finding is currently unknown, but one wonders whether the demonstrated synergism between ethambutol and clarithromycin is a possible reason, since it is expected that the velocity of penetration of antibiotics into the biofilm structure may be delayed by the matrix content, causing both antibiotics to reach the target simultaneously [12].

One of the possible alternative bacterial responses could happen if concentrations above the MIC were to reach the bacteria. This question has been addressed before [22], and it seems that MIC concentrations or concentrations above the MIC trigger similar responses in the bacteria [15]. The same observation was evident in the assays using the MAC 100 strain. Despite increased susceptibility to the majority of the antibiotics, the response to therapy did not change. Clearly, there could be exceptions dependent on the class of antibiotic and the concentration achieved intracellularly or in biofilms. In addition, depending on the organ considered, the bacterial reaction following exposure to antibiotics might vary. Since initial contact between *M. avium* and the antibiotic already induced a transition in RNA and protein synthesis, altering the bacterial response to subsequent antimicrobial molecules, these alterations need to be considered in a clinical situation when administering a treatment regimen [13,14]. Recently, confirmation of this concept was obtained from *Escherichia coli* exposed to ampicillin and ciprofloxacin for 30 min, leading to a transcriptomic response [23].

Infections caused by different pathogens are treated with single or a combination of antibiotics, and recent work has shown that for the most common infections, reducing the period of administration of an active antibiotic from 14 to 7 days can result in similar outcomes compared to much longer treatments [24]. Then, the question that comes to mind is why mycobacterial infections require much longer periods of treatment. The answer, as we currently understand, is complex and multi-layered. Because mycobacteria, in this case, *M. avium*, respond slowly to therapy, this allows for shifts in metabolic pathways, affecting the efficacy of approaches that use the association of different drugs as part of the therapy.

The observation addressed in the current study may indicate that when treating an *M. avium* infection, additional consideration should be given to the sequence in which the therapy is administered. In this study we limited the interval between administration from 5 to 30 min, but in reality, it can be longer, allowing for additional time for the bacterial response.

The limitations of this work are the absence of data showing efficacy in the response to in vivo treatment, and the lack of transcriptomic and proteomic analysis in these tested conditions despite previously referenced information. Future studies will attempt to investigate relevant points regarding the therapy of *M. avium* infections.

## Figures and Tables

**Table 1:** Antimicrobial concentrations effective against *M. avium* 104 and *M. avium* 100.

Antimicrobial/Strain	Minimum Inhibitory Concentration	Minimum Bactericidal Concentration
Rifabutin: 104/100	0.25/0.25 µg/mL	8/4 µg/mL
Clarithromycin: 104/100	2/2 µg/mL	64/16 µg/mL
Ethambutol: 104/100	8/4 µg/mL	64/64 µg/mL

MICs and MBCs were determined as described in Section 2. The values obtained were compared with the serum level achieved by the compound: rifabutin (0.2–0.6 μg/mL), clarithromycin (3–4 μg/mL) and ethambutol (2–6 μg/mL). All of the above compounds reached intracellular concentrations that are greater than the serum concentrations.

**Table 2:** Treatment of *M. avium* biofilms with antimicrobials used in sequence. Effect on survival of *M. avium*.

Treatment ^d^	5 min ^a^	15 min ^a^
CRE	5.0 ± 0.6 × 10^6^	3.6 ± 0.9 × 10^6^
CER	3.0 ± 0.5 × 10^6^	1.2 ± 0.5 × 10^6^
RCE	2.2 ± 0.6 × 10^6^	3.6 ± 0.3 × 10^6^
REC	2.5 ± 0.2 × 10^6^	1.1 ± 0.4 × 10^6^
ERC	1.6 ± 0.8 × 10^6^	2.3 ± 0.6 × 10^6^
ECR	7.7 ± 0.7 × 10^4^ *	2.1 ± 0.3 × 10^5^ *
Same ^b^	2.9 ± 0.5 × 10^4^ *	4.2 ± 0.3 × 10^4^ *
Wildtype ^c^	5.6 ± 0.4 × 10^6^	5.7 ± 0.3 × 10^6^

^a^ Interval of time between addition of antimicrobials. ^b^ Antimicrobials added at the same time. ^c^ Untreated biofilms infected with *M. avium* 104. ^d^ Letter order correlates to antimicrobial treatment order. C: clarithromycin (2 μg/mL); R: rifabutin (0.25 μg/mL); E: ethambutol (8 μg/mL). * *p* < 0.05 compared to the results of the wildtype bacterium.

**Table 3:** Treatment of *M. avium* strain 104-infected macrophages with antimicrobials used in sequence and at the same time. Effect on the survival of intracellular bacteria.

Treatment ^d^	5 min ^a^	15 min	30 min
CRE	4.5 ± 0.2 × 10^4^	6.0 ± 0.2 × 10^4^	6.6 ± 0.7 × 10^4^
CER	6.1 ± 0.6 × 10^4^	4.2 ± 0.6 × 10^4^ *	6.2 ± 0.4 × 10^4^
RCE	5.0 ± 0.8 × 10^4^	7.9 ± 0.5 × 10^4^	7.3 ± 0.5 × 10^4^
REC	6.9 ± 0.2 × 10^4^	4.8 ± 0.2 × 10^4^ *	6.5 ± 0.4 × 10^4^
ERC	3.2 ± 0.9 × 10^4^ *	4.9 ± 0.8 × 10^4^ *	4.1 ± 0.5 × 10^4^ *
ECR	8.3 ± 0.9 × 10^4^	6.0 ± 0.5 × 10^4^ *	3.8 ± 0.6 × 10^4^ *
Same ^b^	4.1 ± 0.4 × 10^4^ *	1.8 ± 0.9 × 10^4^ *	3.3 ± 0.4 × 10^4^ *
Untreated ^c^	7.7 ± 0.6 × 10^4^	1.1 ± 0.8 × 10^5^	4.7 ± 0.6 × 10^5^

^a^. Interval of time between antibiotic exposure to additional antimicrobial. ^b^. Antimicrobials added at the same time. ^c^. Untreated macrophages infected with *M. avium* 104. ^d^. Letter order correlated to antimicrobial treatment order. C: clarithromycin (2 μg/mL). R: rifabutin (0.25 μg/mL). E: ethambutol (8 μg/mL). * *p* < 0.05 compared with wildtype untreated control.

**Table 4:** Treatment of *M. avium* strain 100-infected macrophages with antimicrobials used in sequence and concomitantly. Effect on the survival of intracellular bacteria.

Treatment ^a,d^	5 min	15 min	30 min
CRE	3.0 ± 0.4 × 10^4^	4.2 ± 0.2 × 10^4^	5.1 ± 0.3 × 10^4^
CER	4.0 ± 0.4 × 10^4^	4.2 ± 0.2 × 10^4^	4.5 ± 0.6 × 10^4^
RCE	8.7 ± 0.5 × 10^3^	8.8 ± 0.3 × 10^3^	9.4 ± 0.4 × 10^3^
REC	9.2 ± 0.4 × 10^3^	9.7 ± 0.3 × 10^3^	9.1 ± 0.3 × 10^3^
ERC	5.2 ± 0.5 × 10^3^ *	5.6 ± 0.4 × 10^3^ *	5.6 ± 0.5 × 10^3^ *
ECR	4.9 ± 0.5 × 10^3^ *	4.5 ± 0.6 × 10^3^ *	5.2 + 0.4 × 10^3^ *
Same ^b^	2.9 + 0.3 × 10^3^ *	3.2 + 0.4 × 10^3^ *	2.8 + 0.5 × 10^3^ *
Untreated ^c^	3.7 ± 0.4 × 10^4^	4.1 ± 0.6 × 10^4^	4.4 ± 0.3 × 10^4^

^a^. Interval of time between antibiotic exposure to additional antimicrobial. ^b^. Antimicrobials added at the same time. ^c^. Untreated macrophages infected with *M. avium* 100. ^d^ Letter order correlated to antimicrobial treatment order. C: clarithromycin (2 μg/mL); R: rifabutin (0.25 μg/mL); E: ethambutol (8 μg/mL). * *p* < 0.05 compared with wildtype untreated control.

## Data Availability

The original contributions presented in this study are included in the article. Further inquiries can be directed to the corresponding author.
